# The impact of social capital on physical activity and nutrition in China: the mediating effect of health literacy

**DOI:** 10.1186/s12889-019-8037-x

**Published:** 2019-12-19

**Authors:** Wan-Li Chen, Cheng-Gang Zhang, Zi-Yi Cui, Jing-Ya Wang, Jie Zhao, Ji-Wei Wang, Xian Wang, Jin-Ming Yu

**Affiliations:** 1 Key Lab of Health Technology Assessment of National Health Commission, School of Public Health, Fudan University, No. 130 Dong’an Road, Xuhui District, Shanghai, China; 2 Xuhui District Center for Disease Control and Prevention, No. 50 Yongchuan Road, Xuhui District, Shanghai, China

**Keywords:** Health literacy, Physical activity, Nutrition; mediation analysis

## Abstract

**Background:**

Physical activity and good nutrition are important behavioral factors in promoting health and preventing disease. It is important to understand the factors affecting physical activity and nutrition. The purpose of this study was to explore whether social capital has an effect on physical activity and nutrition, and whether health literacy plays a mediating role between social capital and physical activity as well as nutrition.

**Methods:**

This cross-sectional study was performed in a certain district of Shanghai in March and April 2017. Data was collected using a self-reported questionnaire, which included questions on sociodemographic characteristics, social capital, health literacy and health-promoting lifestyle profile-II. Health-promoting lifestyle profile-II measures the behaviours or habits of physical activity and healthy nutrition. An explore factor analysis of the principal components with varimax rotation was carried out on the social capital scale. Descriptive statistics was used to summarize the sociodemographic of participants. Mediation analysis was performed using the bootstrapping tests to examine whether health literacy mediate the relationship between social capital and physical activity as well as nutrition.

**Results:**

The explore factor analysis results showed that social capital has five dimensions, namely social participation, social support, social network, control over life and feelings about the community. There is a positive correlation between social capital, health literacy, physical activity and nutrition. The correlation coefficient varied from 0.135 to 0.594. Mediation analysis demonstrated health literacy played a partial mediating effect between social capital and physical activity as well as nutrition. In the relationship between physical activity and social capital, the indirect effect of health literacy accounted for 8.20 to 12.65% of the total effect. In the relationship between nutrition and social capital, the mediation effect of health literacy accounted for 4.93 to 12.71% of the total effect.

**Conclusion:**

Social capital can promote physical activity and nutrition by disseminating health information. Enhancing the social capital of residents will help increase physical activity and develop healthy eating habits. Attention should also be paid to the improvement of residents’ health literacy.

## Background

Lack of physical activity is one of the top risk factors for death in the world and a major risk factor for non-communicable diseases such as cardiovascular disease, cancer and diabetes. Physical activity has health benefits for persons of all ages [1]. In adults and the elderly, physical activity can reduce the risk of chronic diseases and cancer. It can also improve bone health and cognitive ability in children and adolescents. For people who are not physically active, even a small increase in physical activity is good for health [2]. According to World health organization, adults aged 18–64 years should do at least 150 min of moderate-intensity physical activity throughout the week, or do at least 75 min of vigorous-intensity physical activity throughout the week, or an equivalent combination of moderate- and vigorous-intensity activity [3]. Taking part in exercise refers to participate in physical exercises (including barehanded or instrumental exercises) once or more in the past year, with the main purpose of strengthening the body and regulating the mind, and achieve a certain intensity of physical activity. In 2014, the proportion of people who regularly participated in physical activities in China was 33.9% (including children and adolescents), and the number of young adults aged 20–49 taking part in exercise is still very small [4]. Nutrition is also related to health. Healthy diet can help reduce many health risks such as overweight, obesity, heart disease and hypertension [5]. According to the China Health and Nutrition Survey, the total energy intake of Chinese residents has declined, but the dietary structure is unreasonable. Oil and salt intake exceeded the recommended level, while beans, dairy products, fruit and vegetable intake remained low [4]. Physical activity and good nutrition are important behavioral factors in promoting health and preventing disease. Therefore, improving the physical activity and nutrition of residents is of great significance for reducing the risk of illness and improving the quality of life.

Recent studies have shown that some social factors (such as social support, social networks, etc.) had a significant impact on physical activity and nutrition [6, 7]. Social support and social networks all belong to the concept of social capital. Whether social capital is an individual or a collective feature is still debated. Individual social capital refers to ‘the ability of actors to secure benefits by virtue of membership in social networks and other social structures’ [8]. Individuals have access to certain benefits and resources that would be impossible without these networks. Putnam, being a proponent of a collective approach, defined social capital as ‘features of social organizations, such as networks, norms and trust that facilitate action and cooperation for mutual benefit’ [9]. Social capital is often viewed as both an individual and a collective feature, although the explicit choice of level of analysis requires different considerations and methods [10]. A bunch of studies in different cultural settings have shown that individuals with more social capital usually have higher level of physical activity [11], better eating habits [12] and lower smoking frequency [13]. A Japanese study reported that higher levels of cognitive social capital are associated with lower likelihood of physical inactivity [14]. A British study showed that there is a positive correlation between individual social capital and consumption of fruits and vegetables [15], and the same conclusions exist in a Swedish study [16]. One study in China showed that social trust was significantly associated with healthy diet, physical activity [17] while having lower neighborhood social capital was associated with insufficient sleep among Japanese adults, particularly in the men [18].

The World Health Organization defines health literacy as ‘the cognitive and social skills which determine the motivation and ability of individuals to gain access to, understand and use information in ways which promote and maintain good health’ [19]. Health literacy affects health-related behaviors because individuals’ ability to make appropriate health-related decisions is related to their skills to find and understand health information [20]. That is to say, one’s ability to make an informed decision is related to his health literacy. Health literacy also reduces the possibility of indulging in health-damaging behaviors such as poor eating habits, smoking and excessive drinking [21]. Recent studies have shown that health literacy is not only related to individual abilities, but also to availability of social resources and the ability of members of social networks [22]. Social capital can not only provide information resources [23], but also become a source of self-efficacy beliefs in finding, understanding and using health information [22], thus having an impact on health literacy.

In order to improve the physical activity and nutrition of residents, it is important to understand the underlying factors that may mediate the link between social capital and physical activity as well as nutrition. Kawachi and Berkman identified diffusion of knowledge on health promotion is one plausible pathway by which social capital exerts a contextual effect on individual health [24]. Therefore, we assume that the relationship between physical activity, nutrition and social capital can be explained by health literacy. The aim of this study was to explore the associated of physical activity and nutrition engagement with social capital, and whether health literacy acts as a mediator in this association.

## Methods

This cross-sectional study was based on data collected from Xuhui district of Shanghai in March and April 2017. Xuhui District is a core urban district of Shanghai. It has a land area of 54.93 km^2^ and a population of 108, 8300 as of 2017, and 33.46% of the residents is above the age of 60. It is a big mixed residential and commercial area. In 2016, the average life expectancy of the registered population in Xuhui District was 84.60 years (82.38 years for males and 86.89 years for females).

### Participants

All participants are the resident population of the city aged 15 to 69 years old (the resident population refers to those who live continuously and live for more than 6 months at the survey site). Stratified cluster random sampling was used in this study. Four streets (towns) were randomly selected from 13 streets (towns) in this district, then one community was selected from the street (town), and 50 households were randomly selected. All the resident population in the household that meets the survey requirements were subject to investigation. A total of 640 people were surveyed and 600 questionnaires were collected. The response rate were 93.8%, and the valid rate was 100%.

Before the participants filled out the self-reported questionnaire, the investigators explained the purpose of the study, the method of data collection and how to complete the questionnaire. They were also informed that their participation was completely anonymous and voluntary. Participants who were unable to complete the questionnaire themselves were assisted by an investigator who read the questions one by one and recorded the answers. All data collection procedures received ethical approval from Ethics Committee of Xuhui District Center for Disease Control and Prevention. A written informed consent was obtained from each participant.

### Measurements

#### Physical activity and nutrition

Physical activity and nutrition was measured using health-promoting lifestyle profile-II (HPLP-II). HPLP-II was developed by Walker et al. [25] to assess residents’ health-promoting lifestyle practices. It consists of 52 items, 6 dimensions (health responsibility, physical activity, nutrition, mental growth, interpersonal relationships and stress management). All items were scored according to a 4-point Likert scale (1 point - never, 2 points - sometimes; 3 points - often; 4 points - always). A score for overall health-promoting lifestyle is obtained by calculating a mean of responses to all 52 items. Subscale scores are determined by calculating the mean of the responses of the subscale items. Higher scores indicate better health lifestyle. The scale shows good reliability and validity in other studies [26]. This study uses physical activity and nutrition dimensions. It measures the behaviours or habits of physical activity and healthy nutrition and not physical activity levels and diet per se.

#### Social capital

Social capital scale is compiled by the expert group and mainly refers to “Social Capital of Organization—World Bank” [27], “Social Capital of Organization” [28], “Social Capital and mental health and well-being” [29], and “Relationship between Community Social Capital and Community Development” [30]. Finally, 24 items were selected to form the initial scale. The original scale consists of six dimensions named social support, social networks, social participation, trust, feelings about the community, and control over life. All items use a 5-point response scale ranging from 1(never) to 5 (always). A high score represents higher level of social capital. The Cronbach’s alpha of this scale is 0.921 and the subscales alphas ranged from 0.765 to 0.923.

#### Health literacy

The 2017 Chinese Residents Health Literacy Monitoring Questionnaire was used to evaluate residents’ health literacy. The scale was developed based on a manual published by the Chinese Ministry of Health in 2015—“Basic Knowledge and Skills of People’s Health Literacy” [31]. It was designed by experts in public health, health education and promotion, and clinical medicine using the Delphi method [32]. The content includes basic knowledge and attitudes, healthy lifestyles and behaviors, and basic skills. There were four types of questions in the scale: true-or-false questions, single-answer questions, multiple-answer questions, and situation questions. For true-or-false and single-answer questions, 1 point was assigned for a correct answer, and 0 point were assigned for an incorrect answer. For multiple-answer questions, 2 points were assigned if the response contained all the correct answers and no incorrect ones, and 0 point were assigned otherwise. For situation questions, participants had to read passages and answer single- or multiple-answer questions about it. Every incorrect answer as well as every unanswered question was scored with 0 point. Residents with a score of ≥52 (65 points) are considered to have basic health literacy. The instrument obtained Cronbach’s alpha of 0.810.

## Data analysis

Data was analyzed with SPSS version 22.0 and PROCESS version 3.2. Descriptive statistics were used to summarize the sociodemographic of participants. Explore factor analysis (EFA) of the principal components with varimax rotation was carried out on the social capital scale. An eigenvalue > 1 was used as a cut-off point to determine the applicability of the pre-designed factors of the social capital. Items with factor loading > 0.5 and conceptual relevance were used as criteria for retaining in a factor. Items with loadings > 0.4 on two or more factors were excluded.

Mediation analysis was performed using the bootstrapping tests [33] to examine whether health literacy mediate the relationship between social capital and physical activity as well as nutrition. We presented the gender-stratified and age catogories-stratified results. Multicollinearity between physical activity, nutrition, social capital and health literacy was assessed by performing Pearson correlations. Highly correlated variables that indicate multicollinearity(r > 0.90), or variables that were not correlated with either physical activity, nutrition or social capital, were excluded from the subsequent mediation analyses based on recommendations for multivariate analyses [34]. The significance level of this study was *P* ≤ 0.05.

**Fig. 1:**
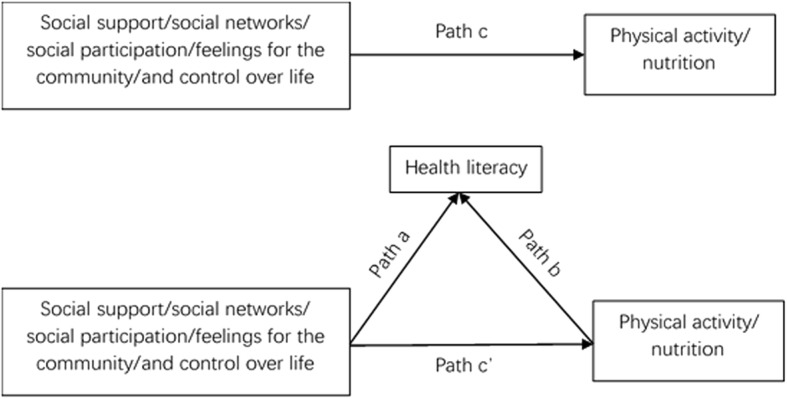
Example of the mediation model tested in this study

The mediation effect analysis needs to meet the following conditions: 1) social capital was significantly associated with physical activity and nutrition (total effect; c path, Fig. 1); 2) social capital was significantly associated with health literacy; 3) controlling for social capital, health literacy was significantly associated with physical activity and nutrition (b paths); and 4) the relationship between social capital and physical activity as well as nutrition was reduced (direct effect, c’ path) when controlling for health literacy (indirect effect, a x b), with the 95% confidence interval (CI) for the indirect effect of each proposed mediating variable outside 0.

## Results

Table 1 presents the sociodemographic characteristics of participants in this study. Among the 600 participants, 275 were males, accounting for 45.8%. Over half of the participants were between the ages of 60 and 69, accounting for 41.8%. The most common educational attainment was college or university graduate.

**Table 1:** Sociodemographic characteristics of main study participants (*n* = 600)

Characteristics	N(%)
Sex	
Male	275 (45.8)
Female	325 (54.2)
Age	
< 45	182 (30.3)
45–59	167 (27.8)
60–69	251 (41.8)
Educational attainment	
Junior high school and below	176 (29.3)
Senior high school	193 (32.2)
college or university graduate	231 (38.5)

Table 2 presents the factor loadings based on EFA, Cronbach’s α, score mean and standard deviation for these scales and items. The Kaiser-Meyer-Olkin measure (KMO value of 0.900) and Bartlett’s test (*P* < 0.001) confirmed the adequacy for the EFA. EFA yielded five factors with eigenvalues> 1 that accounted for 78.24% of total variance. Six items were deleted in light of the criteria mentioned above. After analyzing and categorizing the meanings of the factors and the items they contain, the five dimensions are named for social support, social networks, social participation, feelings about the community, and control over life.

**Table 2:** Factor loading. Reliability coefficient. Mean and SD

	Item factor loading	reliability coefficient(α)	Mean (SD)
Social participation		0.890	
Participate in activities organized by the community (streets, neighborhood committees, local hospitals, etc.)	0.865		3.13 (1.10)
Participate in volunteer activity.	0.887		3.07 (1.14)
Participate in activities organized by social groups, civil associations, etc.	0.804		2.78 (1.14)
Participate in leisure activities (such as cultural and sporting activities, tourism, etc.).	0.676		3.35 (0.92)
Social networks		0.765	
Stay with relatives.	0.818		3.64 (0.78)
Stay with friends.	0.811		3.68 (0.80)
Social support		0.817	
Have a sense of camaraderie with others.	0.690		3.51 (0.88)
Get help from others when you need it.	0.750		3.61 (0.77)
You can feel that others care about you.	0.771		3.68 (0.75)
Someone will take the initiative to share his or her social experience and joy of life.	0.756		3.62 (0.78)
Someone is willing to listen to your thoughts and troubles.	0.779		3.66 (0.79)
Someone will listen to your opinions when deciding or discussing things.	0.748		3.68 (0.79)
Control over life		0.895	
Have self-esteem	0.888		4.29 (0.68)
Have self-confidence	0.893		4.26 (0.70)
Satisfied with your own life	0.648		4.13 (0.75)
feelings about the community		0.923	
Satisfied with the environment of the community in which you live.	0.891		3.93 (0.80)
Satisfied with the public facilities of the community in which you live.	0.882		3.93 (0.77)
Have a sense of security in the neighborhood and on the surrounding roads.	0.832		3.92 (0.81)

Table 3 presents the relationship between variables. There is a positive correlation between social capital, health literacy, physical activity and nutrition. The correlation coefficient varied from 0.135 to 0.594. All correlation coefficients were below 0.90 indicating that there is no multicollinearity among these variables.

**Table 3:** Correlation between social capital, health literacy, physical activity and nutrition

	1	2	3	4	5	6	7	8
1.social participation	1							
2. social networks	0.500^**^	1						
3.social support	0.549^**^	0.482^**^	1					
4.control over life	0.257^**^	0.337^**^	0.564^**^	1				
5.feelings about the community	0.212^**^	0.302^**^	0.486^**^	0.571^**^	1			
6.health literacy	0.228^**^	0.135^**^	0.293^**^	0.161^**^	0.164^**^	1		
7.physical activity	0.556^**^	0.436^**^	0.594^**^	0.353^**^	0.377^**^	0.326^**^	1	
8.nutrition	0.303^**^	0.316^**^	0.460^**^	0.344^**^	0.504^**^	0.229^**^	0.593^**^	1

*P < 0.05;

**P < 0.01;

All *P* values are two-tailed

Table 4 presents the mediating effect of health literacy. Five dimensions of social capital are significantly associated with physical activity (path c: social support, B = 0.768, *p* < 0.001; social network, B = 1.572, *p* < 0.001; social participation, B = 0.758, *p* < 0.001; control over life, B = 0.933, *p* < 0.001; feelings about the community, B = 0.868, *p* < 0.001) and nutrition (path c: social support, B = 0.510, *p* < 0.001; social network, B = 0.976, *p* < 0.001; social participation, B = 0.354, *p* < 0.001; control over life, B = 0.779, *p* < 0.001; feelings about the community, B = 0.994, *p* < 0.001) and therefore are predictors of good habits of physical activity and nutrition. Health literacy partially mediate the association of physical activity and nutrition with social capital. In the relationship between physical activity and social capital, the indirect effect of health literacy accounted for 8.20 to 12.63% of the total effect. In the relationship between nutrition and social capital, the mediation effect of health literacy accounted for 4.93 to 12.71% of the total effect.

**Table 4:** Gender-stratified analysis of mediation effect of health literacy

Model	Path	Total				Male				Female			
		β	95% CI	*p*-value	Proportion mediated	β	95% CI	*p*-value	Proportion mediated	β	95% CI	*p*-value	Proportion mediated
social participation to physical activity	Total effect (c)	0.758	(0.667, 0.849)	< 0.001		0.798	(0.654, 0.942)	< 0.001		0.735	(0.615, 0.856)	< 0.001	
	Direct effect (c’)	0.693	(0.602, 0.784)	< 0.001		0.721	(0.580, 0.863)	< 0.001		0.682	(0.561, 0.503)	< 0.001	
	Indirect effect (a x b)	0.065	(0.035, 0.100)		8.58%	0.077	(0.027, 0.139)		9.65%	0.054	(0.019, 0.097)		7.35%
social networks to physical activity	Total effect (c)	1.572	(1.311, 1.833)	< 0.001		1.515	(1.107, 1.923)	< 0.001		1.601	(1.262, 1.941)	< 0.001	
	Direct effect (c’)	1.439	(1.188, 1.691)	< 0.001		1.368	(0.979, 1.757)	< 0.001		1.490	(1.160, 1.821)	< 0.001	
	Indirect effect (a x b)	0.133	(0.045, 0.239)		8.46%	0.147	(−0.002, 0.322)		–	0.112	(0.004, 0.229)		7.00%
social support to physical activity	Total effect (c)	0.768	(0.685, 0.852)	< 0.001		0.822	(0.695, 0.948)	< 0.001		0.721	(0.609, 0.834)	< 0.001	
	Direct effect (c’)	0.706	(0.620, 0.791)	< 0.001		0.749	(0.615, 0.883)	< 0.001		0.671	(0.558, 0.784)	< 0.001	
	Indirect effect (a x b)	0.063	(0.034, 0.099)		8.20%	0.073	(0.016, 0.139)		8.88%	0.050	(0.017, 0.091)		6.93%
Control over life to physical activity	Total effect (c)	0.933	(0.734, 1.131)	< 0.001		0.976	(0.675, 1.278)	< 0.001		0.899	(0.635, 1.163)	< 0.001	
	Direct effect (c’)	0.815	(0.622, 1.008)	< 0.001		0.787	(0.489, 1.086)	< 0.001		0.839	(0.585, 1.094)	< 0.001	
	Indirect effect (a x b)	0.118	(0.055, 0.189)		12.65%	0.189	(0.080, 0.325)		19.36%	0.060	(−0.011, 0.144)		–
feelings about the community to physical activity	Total effect (c)	0.868	(0.697, 1.039)	< 0.001		0.731	(0.459, 1.002)	< 0.001		0.983	(0.766, 1.200)	< 0.001	
	Direct effect (c’)	0.765	(0.599, 0.931)	< 0.001		0.610	(0.349, 0.872)	< 0.001		0.898	(0.684, 1.111)	< 0.001	
	Indirect effect (a x b)	0.103	(0.046, 0.168)		11.87%	0.12	(0.027, 0.238)		16.42%	0.085	(0.024, 0.164)		8.65%
social participation to nutrition	Total effect (c)	0.354	(0.265, 0.444)	< 0.001		0.376	(0.243, 0.509)	< 0.001		0.332	(0.207, 0.457)	< 0.001	
	Direct effect (c’)	0.309	(0.219, 0.400)	< 0.001		0.320	(0.187, 0.453)	< 0.001		0.298	(0.171, 0.425)	< 0.001	
	Indirect effect (a x b)	0.045	(0.022, 0.073)		12.71%	0.056	(0.018, 0.105)		14.89%	0.034	(0.006, 0.071)		10.24%
social networks to nutrition	Total effect (c)	0.976	(0.740, 1.211)	< 0.001		0.880	(0.533, 1.226)	< 0.001		1.038	(0.714, 1.362)	< 0.001	
	Direct effect (c’)	0.896	(0.663, 1.130)	< 0.001		0.784	(0.445, 1.123)	< 0.001		0.979	(0.655, 1.302)	< 0.001	
	Indirect effect (a x b)	0.079	(0.025, 0.145)		8.09%	0.096	(−0.002, 0.217)		–	0.059	(0.001, 0.135)		5.68%
social support to nutrition	Total effect (c)	0.510	(0.431, 0.589)	< 0.001		0.519	(0.405, 0.633)	< 0.001		0.498	(0.388, 0.609)	< 0.001	
	Direct effect (c’)	0.477	(0.394, 0.559)	< 0.001		0.472	(0.350, 0.593)	< 0.001		0.476	(0.363, 0.589)	< 0.001	
	Indirect effect (a x b)	0.034	(0.009, 0.061)		6.67%	0.048	(0.002, 0.100)		9.25%	0.023	(−0.003, 0.053)		–
Control over life to nutrition	Total effect (c)	0.779	(0.609, 0.950)	< 0.001		0.728	(0.481, 0.975)	< 0.001		0.826	(0.590, 1.063)	< 0.001	
	Direct effect (c’)	0.714	(0.544, 0.885)	< 0.001		0.614	(0.364, 0.864)	< 0.001		0.796	(0.561, 1.030)	< 0.001	
	Indirect effect (a x b)	0.065	(0.028, 0.114)		8.34%	0.114	(0.037, 0.217)		15.66%	0.031	(−0.006, 0.081)		–
feelings about the community to nutrition	Total effect (c)	0.994	(0.858, 1.131)	< 0.001		0.933	(0.730, 1.136)	< 0.001		1.048	(0.863, 1.233)	< 0.001	
	Direct effect (c’)	0.946	(0.809, 1.082)	< 0.001		0.868	(0.668, 1.069)	< 0.001		1.015	(0.828, 1.202)	< 0.001	
	Indirect effect (a x b)	0.049	(0.019, 0.085)		4.93%	0.065	(0.014, 0.133)		6.97%	0.033	(0.001, 0.077)		3.15%

We found different mediation effects after gender stratifying. For male, health literacy played a partial mediating role in the association of social participation, social support, control over life, and feelings about the community with physical activity as well as nutrition. For female, health literacy partially mediated the effects of social participation, social network, social support and feelings about the community on physical activity, and the effects of social participation, social network and feelings about the community on nutrition.

In the age categories-stratified analysis, health literacy mediated the association of five dimensions of social capital with physical activity and nutrition among residents under the age of 45. Health literacy played a partial mediating role in the association of social participation, social support, control over life, and feelings about the community with physical activity among residents aged 45–59. Among residents aged 60–69, health literacy mediated the relationship between social participation, social support and physical activity, and the relationship between social participation and nutrition. The results were presented in Table 5.

**Table 5:** Age categories-stratified analysis of mediation effect of health literacy

Model	Path	< 45 years old				45–59 years old				60–69 years old			
		β	95% CI	*p*-value	Proportion mediated	β	95% CI	*p*-value	Proportion mediated	β	95% CI	*p*-value	Proportion mediated
social participation to physical activity	Total effect (c)	0.803	(0.624, 0.982)	< 0.001		0.636	(0.455, 0.816)	< 0.001		0.819	(0.682, 0.955)	< 0.001	
	Direct effect (c’)	0.708	(0.526, 0.891)	< 0.001		0.537	(0.353, 0.720)	< 0.001		0.767	(0.633, 0.902)	< 0.001	
	Indirect effect (a x b)	0.095	(0.037, 0.169)		11.83%	0.099	(0.038, 0.176)		15.57%	0.051	(0.012, 0.105)		6.23%
social networks to physical activity	Total effect (c)	1.551	(1.004, 2.097)	< 0.001		1.303	(0.769, 1.837)	< 0.001		1.691	(1.332, 2.051)	< 0.001	
	Direct effect (c’)	1.259	(0.716, 1.801)	< 0.001		1.131	(0.622, 1.640)	< 0.001		1.61	(1.266, 1.955)	< 0.001	
	Indirect effect (a x b)	0.292	(0.114, 0.511)		18.83%	0.172	(−0.076, 0.422)		–	0.081	(−0.027, 0.212)		–
social support to physical activity	Total effect (c)	0.670	(0.509, 0.831)	< 0.001		0.809	(0.643, 0.976)	< 0.001		0.793	(0.671, 0.915)	< 0.001	
	Direct effect (c’)	0.578	(0.410, 0.747)	< 0.001		0.728	(0.549, 0.908)	< 0.001		0.744	(0.622, 0.866)	< 0.001	
	Indirect effect (a x b)	0.091	(0.036, 0.158)		13.58%	0.081	(0.006, 0.161)		10.01%	0.049	(0.013, 0.102)		6.18%
Control over life to physical activity	Total effect (c)	0.780	(0.412, 1.149)	< 0.001		1.069	(0.673, 1.464)	< 0.001		0.935	(0.637, 1.232)	< 0.001	
	Direct effect (c’)	0.598	(0.238, 0.957)	0.001		0.87	(0.479, 1.260)	< 0.001		0.875	(0.590, 1.161)	< 0.001	
	Indirect effect (a x b)	0.183	(0.062, 0.343)		23.46%	0.199	(0.071, 0.372)		18.62%	0.059	(−0.033, 0.160)		–
feelings about the community to physical activity	Total effect (c)	0.695	(0.373, 1.018)	< 0.001		0.917	(0.594, 1.240)	< 0.001		0.914	(0.650, 1.179)	< 0.001	
	Direct effect (c’)	0.570	(0.260, 0.880)	< 0.001		0.737	(0.413, 1.062)	< 0.001		0.846	(0.591, 1.102)	< 0.001	
	Indirect effect (a x b)	0.125	(0.014, 0.258)		17.99%	0.18	(0.062, 0.357)		19.63%	0.068	(−0.008, 0.160)		–
social participation to nutrition	Total effect (c)	0.341	(0.156, 0.526)	< 0.001		0.272	(0.102, 0.443)	0.002		0.405	(0.272, 0.538)	< 0.001	
	Direct effect (c’)	0.240	(0.051, 0.429)	0.013		0.2	(0.024, 0.375)	0.026		0.379	(0.244, 0.514)	< 0.001	
	Indirect effect (a x b)	0.102	(0.041, 0.180)		29.91%	0.072	(0.020, 0.142)		26.47%	0.026	(0.001, 0.063)		6.42%
social networks to nutrition	Total effect (c)	1.000	(0.490, 1.509)	< 0.001		0.675	(0.199, 1.150)	0.006		1.1	(0.779, 1.421)	< 0.001	
	Direct effect (c’)	0.760	(0.249, 1.271)	0.004		0.567	(0.098, 1.035)	0.018		1.061	(0.743, 1.380)	< 0.001	
	Indirect effect (a x b)	0.239	(0.088, 0.428)		23.90%	0.108	(−0.048, 0.278)		–	0.039	(−0.013, 0.113)		–
social support to nutrition	Total effect (c)	0.394	(0.235, 0.552)	< 0.001		0.504	(0.344, 0.663)	< 0.001		0.576	(0.466, 0.687)	< 0.001	
	Direct effect (c’)	0.306	(0.139, 0.472)	< 0.001		0.456	(0.282, 0.630)	< 0.001		0.56	(0.448, 0.673)	< 0.001	
	Indirect effect (a x b)	0.088	(0.033, 0.152)		22.34%	0.048	(−0.029, 0.114)		–	0.016	(−0.011, 0.047)		–
Control over life to nutrition	Total effect (c)	0.833	(0.509, 1.156)	< 0.001		0.609	(0.256, 0.963)	0.001		0.843	(0.597, 1.089)	< 0.001	
	Direct effect (c’)	0.704	(0.382, 1.026)	< 0.001		0.485	(0.127, 0.842)	0.008		0.815	(0.572, 1.059)	< 0.001	
	Indirect effect (a x b)	0.129	(0.039, 0.257)		15.49%	0.125	(0.030,, 0.258)		20.53%	0.027	(−0.013, 0.088)		–
feelings about the community to nutrition	Total effect (c)	0.954	(0.686, 1.223)	< 0.001		0.956	(0.693, 1.219)	< 0.001		1.031	(0.827, 1.235)	< 0.001	
	Direct effect (c’)	0.870	(0.607, 1.134)	< 0.001		0.882	(0.610, 1.154)	< 0.001		1.003	(0.800, 1.207)	< 0.001	
	Indirect effect (a x b)	0.084	(0.009, 0.170)		8.81%	0.074	(0.001, 0.181)		7.74%	0.027	(−0.004, 0.075)		–

## Discussion

This cross-sectional study examined the relationship between social capital and health-promoting lifestyle. It also explores if health literacy acts as a mediator in this association. EFA yielded five dimensions named social support, social networks, social participation, feelings about the community, and control over life. The results demonstrate that social capital has a positive impact on physical activity and nutrition. Health literacy mediates the relationship between social capital and physical activity as well as nutrition.

Social capital can directly influence physical activity and nutrition. Social support is one of the important social psychological factors affecting the physical activities of residents. It also has been demonstrated that good social support is associated with higher quality diet [35]. Social support from friends and family in the form of offering encouragement, establishing connections and providing accountability has been shown to help improve adherence for a wide variety of health behaviors, including a healthier diet [36] and exercising more [37, 38]. Social networks may influence physical activity by providing social support. A study in Brazil showed that people with four or more friends had significantly different levels of physical activity than people without friends [39]. In a large, observational study of more than 20,000 adults over age 50, being single, widowed, or having less frequent contact with friends was associated with less variety of fruit and vegetable intake [40]. Our research supports these conclusions. Besides, social network members’ lifestyle can influence an individual’s choices. Research shows at least one social network member who encourages a healthy diet is associated with motivation to consume more fruits and vegetables [41]. Social participation also has a positive impact on physical activity and nutrition, as social participation can extend the social networks of residents. Participation in running clubs and nutrition education class can directly influence members’ physical activity level and healthy diet. Social groups, even those whose focus is not directly about health (e.g. religious organizations and other interest groups), tend to provide opportunities for its members to know and stay alert about health-related issues [42]. However, a person’s social groups may engage in unhealthful behavior, such as smoking or heavy drinking. In such circumstances, pressures to conform to group standards may produce unhealthy behavior [43]. Previous studies have shown that physical activity and healthy diet can improve life satisfaction [44, 45]. The impact of life satisfaction on physical activity and healthy diet needs further study. The influence of feelings about community on physical activity and nutrition may be attributed to the community-surrounding environment. The convenience of the surrounding environment and the better accessibility of neighborhood parks and markets has been related to the increase in physical activity [46] and consumption of vegetables and fruits [47].

Health literacy plays a partial mediating role in the association of physical activity and nutrition with social capital. The impact of social capital on physical activity and nutrition can be partly explained by health literacy. Social capital can provide a better social environment for access to health information. Larger social networks can expand the source of information and promote the spread of health information among network members. Participation in various organizations and activities can make connection with more group members and extend social networks. Family, relatives, or friends are often the first consulted regarding health concerns. Informational support may assist people in accessing and comprehending health information [48]. The feeling of belonging in the community may lead to a closer connection among members. Social capital also can be a source of self-efficacy belief for finding, understanding, and using health information [22]. Therefore, social capital can promote the improvement of health literacy. People with better health literacy may understand the importance of physical activity and a healthy diet, and become more involved in healthy lifestyles. A lifeline cohort study showed that higher health literacy is associated with positive health behaviors, including regular diet and adequate physical activity [49]. At present, there are few studies examining the mediating role of health literacy in social capital and health behavior. There are some similar studies indicated that health literacy mediated the relationship between educational attainment and health behavior, especially in relation to physical activity and diet [50]. Another study found that self-efficacy mediated the relationship between social support and physical activity. Social support can influence adolescent girls’ physical activity through enjoyment, self-efficacy, overcoming barriers to physical activity, motivation, and performance improvements, as well as enabling physical activity [51]. Social capital may affect physical activity and nutrition through other factors. Further research is needed to understand how social capital influence physical activity and nutrition.

In the mediation analysis stratified by gender, health literacy did not mediate the association of physical activity and nutrition with social network in males. It may be because men are less interested and less frequent than women in health-related information exchange with social network members. Health literacy is not a mediator of social support and nutrition in females. This may contradicts the previous statement. It may be because social support such as informational support can directly help women adopt healthy nutrition behavior without the mediation of health literacy. In the age categories-stratified mediation analysis, health literacy only mediates the association of physical activity and nutrition with social participation among residents aged 60–69.

There are some limitations in this study. First, the cross-sectional setting of our study does not allow causal inferences. Second, older population account for a higher proportion of participants, which do not represent the proportion of the general population in Shanghai. Besides, all participants are from a region of Shanghai that is an economically developed city of China. Social capital may be differ from regions. Cluster sampling is prone to biases and may affects the representativeness of the sample and the generalizability of results. Moreover, the mediation analysis of individual-level, ignoring the clustering, and may increase type I error. Finally, the social capital scale used in this study is self-compiled. Its reliability and validity need to be further verified.

## Conclusion

This study found that health literacy plays a mediating role in the impact of social capital on physical activity and nutrition. Social capital can promote healthy lifestyles by disseminating health information. Enhancing the social capital of residents will help increase physical activity and improve nutritional status. Moreover, attention should also be paid to the improvement of residents’ health literacy.

## Data Availability

The datasets used and/or analyzed during the current study are available from the corresponding author on reasonable request.

## Abbreviations

EFA: Explore Factor Analysis
